# Paradoxical Effect of Nonalcoholic Red Wine Polyphenol Extract, Provinols™, in the Regulation of Cyclooxygenases in Vessels from Zucker Fatty Rats (*fa*/*fa*)

**DOI:** 10.1155/2017/8536910

**Published:** 2017-06-04

**Authors:** Abdelali Agouni, Hadj Ahmed Mostefai, Anne-Hélène Lagrue, Martina Sladkova, Philippe Rouet, Franck Desmoulin, Olga Pechanova, Maria Carmen Martínez, Ramaroson Andriantsitohaina

**Affiliations:** ^1^INSERM U1063, Stress Oxydant et Pathologies métaboliques, Université d'Angers, Université Bretagne Loire, Angers, France; ^2^Pharmaceutical Science Section, College of Pharmacy, Qatar University, Doha, Qatar; ^3^Institute of Normal and Pathological Physiology, Slovak Academy of Sciences, Bratislava, Slovakia; ^4^UMR UT3 CNRS 5288, Team Evolutionary Medicine, Obesity and Heart Failure: Molecular and Clinical Investigations, Avenue Jean Poulhes, Toulouse, France; ^5^ToNIC, Toulouse NeuroImaging Center, Université de Toulouse, Inserm, UPS, Toulouse, France; ^6^CHU, Angers, France

## Abstract

The aim of this work was to study the vascular effects of dietary supplementation of a nonalcoholic red wine polyphenol extract, Provinols, in Zucker fatty (ZF) obese rats. ZF or lean rats received diet supplemented or not with Provinols for 8 weeks. Vasoconstriction in response to phenylephrine (Phe) was then assessed in small mesenteric arteries (SMA) and the aorta with emphasis on the contribution of cyclooxygenases (COX). Although no difference in vasoconstriction was observed between ZF and lean rats both in SMA and the aorta, Provinols affected the contribution of COX-derived vasoconstrictor agents. The nonselective COX inhibitor, indomethacin, reduced vasoconstriction in vessels from both groups; however, lower efficacy was observed in Provinols-treated rats. This was associated with a reduction in thromboxane-A2 and 8-isoprostane release. The selective COX-2 inhibitor, NS398, reduced to the same extent vasoconstriction in aortas from ZF and Provinols-treated ZF rats. However, NS398 reduced response to Phe only in SMA from ZF rats. This was associated with a reduction in 8-isoprostane and prostaglandin-E release. Paradoxically, Provinols decreased COX-2 expression in the aorta, while it increased its expression in SMA. We provide here evidence of a subtle and paradoxical regulation of COX pathway by Provinols vessels from obese rats to maintain vascular tone within a physiological range.

## 1. Introduction

The incidence of obesity has increased significantly worldwide in the past decade, reaching epidemic proportions. Obesity is commonly associated with comorbidities (such as hypertension, dyslipidaemia, and diabetes) and with an increased risk of premature death [1]. Obesity, in particular abdominal obesity, was shown to be a primary contributor to acquired insulin resistance, as increasing adiposity is correlated with impaired insulin action [2, 3]. Obesity is also associated with a broad inflammatory response, particularly, but not exclusively, in vessels [4, 5].

There are several inflammatory mediators that impact the vascular function in different animal models of obesity and metabolic syndrome, such as cyclooxygenase- (COX-) derived prostanoid derivatives [4–6]. Arachidonic acid is released by membrane phospholipids through phospholipase A2 cleavage; it can be metabolized by COX pathway into prostaglandins and thromboxane A2 (TXA2) [7]. COX exists in two major isoforms (COX-1 and COX-2) and one variant (COX-3) [8]. COX-1 is found in all tissues, while COX-2 is known as an inducible enzyme that produces, in most cases, large amounts of prostaglandins. COX-2 is constitutively expressed in only a few sites, such as parts of the kidney and central nervous system, but is highly upregulated and active at the sites of inflammation including vascular smooth muscle cells and adipose tissue. It is known that many prostanoid derivatives have specific vasoactive properties, thereby contributing to the local regulation of vascular function [9].

Polyphenols constitute one of the most numerous and ubiquitously distributed group of plant secondary metabolites, present in all plants that are commonly consumed in the Mediterranean diet, including grains, legumes, fruits, vegetables, extra virgin olive oil, and red wine [10, 11]. Epidemiological studies report an inverse association between dietary polyphenol consumption and mortality from cardiovascular diseases [12, 13]. Furthermore, red wine-derived polyphenols have been reported to improve cardiovascular function in various animal models of cardiovascular diseases and metabolic disturbances. For instance, red wine polyphenols were reported to improve endothelial function and normalise blood pressure in NO-deficient hypertensive rats [14], in spontaneously hypertensive rats [15] and deoxycorticosterone acetate- (DOCA-) salt-induced hypertensive rats [16]. In addition, red wine polyphenols were found to protect against cardiac ischemia [17], stroke [18], and improved cardiac function and endothelial function in obese rats [19]. Dietary polyphenols have also been found to inhibit cellular enzymes, such as phospholipase A2 and COX, in order to reduce arachidonic acid and prostaglandin production, thus exerting an important anti-inflammatory action. Polyphenolic compounds extracted from red wine were able to modulate COX-2 activity and gene expression in different cell types [20–22].

We have previously reported that dietary red wine polyphenol extract, Provinols, improves endothelial function in the obese Zucker fatty (ZF) rat, a common animal model of obesity and metabolic syndrome, by reducing oxidative stress and enhancing nitric oxide (NO) bioavailability [19]. However, the effects of Provinols on vascular inflammation and vasoreactivity have not yet been studied. The aim of the present work was therefore to investigate the vascular effects of a dietary supplementation of Provinols in ZF rats, with respect to the COX pathway regulation.

## 2. Materials and Methods

### 2.1. Animals and Experimental Protocol

All animal studies were carried out using approved institutional protocols and conform the *Guide for the Care and Use of Laboratory animals* published by US National Institutes of Health (NIH Publication number 85-23, revised 1996). The study involved 12 Zucker fatty (ZF) rats and 12 of their lean nonobese rats (Charles River, L'Arbresle, France); all of which received, for eight weeks, either a control diet or a diet containing a dose of 20 mg/kg/day of Provinols (Société Française de Distilleries; Vallon Pont d'Arc, France), a nonalcoholic red wine polyphenol extract. The dose of Provinols given to animals was described to be able to induce several cardiovascular protective effects, such as the improvement of endothelial function [23], the prevention of the increase in blood pressure in NO-deficient hypertensive rats [14], and the protection against cardiac ischemia [17], stroke [18], and improved cardiac function and endothelial function in obese rats [19]. The eight-week treatment has been reported to protect against the deleterious effects of hypertension [24] and chronic stress exposure [25] and obesity [19].

### 2.2. Vascular Reactivity

Aortic and small mesenteric arteries (SMA) were obtained from ZF or lean rats having or having not received Provinols, which were then cleaned from connective tissue and cut into rings of 1.5–2 mm long. Vessel rings were then mounted on a wire myograph (Danish MyoTechnology, Aarhus, Denmark) filled with physiological salt solution (PSS) as described previously [19, 26, 27]. The concentration-response curves were constructed by cumulative application of phenylephrine (Phe, 1 nmol/L to 10 *μ*mol/L; Sigma-Aldrich, St. Quentin, Fallavier, France) to vessels with functional endothelium in the presence or absence of a given inhibitor: the NO synthase inhibitor N*^ω^*-nitro-L-arginine methyl ester (L-NAME, 100 *μ*mol/L; Sigma-Aldrich), the selective COX-2 inhibitor N-(2-cyclohexyloxy-4-nitrophenyl) methanesulfonamide (NS398, 10 *μ*mol/L; Sigma-Aldrich), or indomethacin, the nonselective COX inhibitor (INDO, 100 *μ*mol/L). All inhibitors were used at their maximal active concentrations at which they inhibit the release of either NO from all isoforms of NO synthases (L-NAME), metabolites from COX-2 isoforms (NS398), or metabolites from COX (INDO) in blood vessels, as reported in our previous studies [26].

### 2.3. Determination of Prostanoid Production

Vessels with endothelium were treated with Phe (10 *μ*mol/L, 30 minutes, 37°C). The medium was next collected. Then, TXA2, prostaglandin E metabolites, prostacyclin, and total 8-isoprostane were measured by enzyme immunoassays kits (Cayman Chemicals, Ann Arbor, Michigan). The concentration of prostanoids was expressed as pg/mL/mg of dry weight (dw) tissue [28].

### 2.4. Western Blotting

Aortas and SMA vessels were dissected, homogenized, and lysed. Proteins (80 *μ*g) were separated on 10% SDS-PAGE. Blots were probed with anticyclooxygenase- (COX-) 1 (Santa Cruz Biotechnology, Santa Cruz, CA), anti-COX-2 (BD Biosciences, San Jose, CA), and anti-NF-*κ*B p65 (Abcam, Paris, France) antibodies. A monoclonal mouse *β*-actin antibody (Sigma-Aldrich) was used at 1/5000 dilution for visualization of protein gel loading. The membranes were then washed at least three times in Tris buffer solution containing 0.05% Tween and incubated for 1 hour per wash at room temperature, with the appropriate horseradish peroxidase- (HRP-) conjugated secondary antibody (Amersham). The protein antibody complexes were detected by ECL plus (Amersham) according to the protocol of the manufacturer as previously done [29, 30].

### 2.5. Staining and Imaging by Confocal Microscopy

Vessels with endothelium were frozen and cut into 7 *μ*m sections. Fixed sections were incubated (2 hours) in blocking buffer (5% nonfat dry milk in phosphate-buffered saline). After three washes, tissue sections were incubated overnight with a polyclonal NF-*κ*B p65 antibody (1 : 100). After three washes, sections were incubated (1 hour) with murine Alexa fluor-488-labeled antibody (1 : 100). After washes, vessel sections were mounted on glass slides and a Solamere confocal equipment with a DLS-300 laser mounted on a Nikon Eclipse TE 2000-S inverted microscope was used for the optical sectioning of the tissue. Digital image recording was performed using the QED in vivo software.

### 2.6. Carotid Tissue Dual Phase Metabolite Extraction

#### 2.6.1. Extraction Protocol

Frozen sections of carotids (*n* = 10, 25 ± 8 mg) were powdered in liquid N_2_ with a mortar and pestle and, then, immediately extracted using a dual phase extraction method [31]. Briefly, frozen tissue fragments were homogenized for 20 seconds in ice-cold solvents (purex-analytical-grade) methanol and chloroform in a ratio of 2 : 1 (1.5 mL) by using an Ultra-Turrax homogenizer. After 5 minutes in contact with the first solvents at 4°C, 0.5 mL chloroform and 0.5 mL distilled water were added and homogenized. The samples were then centrifuged at 2000*g* for 30 minutes. The organic fraction was collected. Vials were sealed under a flow of Ar and maintained at −20°C. Prior analysis, lipid extracts were dried off and dissolved in 400 *μ*L of chloroform.

#### 2.6.2. Phospholipid Analysis

The lipid extract (200 *μ*L from the total 400 *μ*L) was dried and dissolved in 50 *μ*L of chloroform/methanol (1 : 1, v/v); 20 *μ*L was analysed by HPLC on a Summit DIONEX system using an Uptisphere 6OH (5 *μ*mol/L) 250 × 2 mm column. The flow rate was 0.25 mL/minute, and the column temperature was kept at 25°C during all runs. A light-scattering detector was used for the detection (Polymer Laboratories PL-ELS2100, Church Stretton, United Kingdom). The evaporator and nebulisator temperature was kept, respectively, at 50 and 80°C, and the nitrogen flow was 1.8 mL/minute. A binary solvent system was used with (A): hexane/isopropanol (82 : 18, v/v) and (B): isopropanol/water (85 : 15, v/v) in the presence of triethylamine (0.014%, v/v) and acetic acid (0.5%, v/v). The gradient profile was started at 5% B for 5 minutes, moved to 35% B in 25 minutes, and then to 85% B in 8 minutes. Finally, the gradient was returned to the starting conditions in 10 minutes. The column was equilibrated for 10 minutes before the next run.

#### 2.6.3. Fatty Acid Analysis

Glyceryl triheptadecanoate (2 *μ*g) as internal standard and hexane (1 mL) was added to the lipid extract (80 *μ*L from the total 400 *μ*L). Transmethylation was performed for 1 hour in boron trifluoride methanol solution 14% (1 mL, Sigma-Aldrich) at 55°C. After the addition of water (1 mL) to the crude, fatty acid methyl esters (FAME) were extracted with hexane (3 mL), dried by evaporation, and dissolved in ethyl acetate (20 *μ*L). FAME (1 μL) were analysed by gas-liquid chromatography [32] on a 5890 Hewlett Packard system using a Famewax RESTEK-fused silica capillary columns (30 m × 0.32 mm i.d., 0.25 mm film thickness). Oven temperature was programmed from 110°C to 220°C at a rate of 2°C per min, and the carrier gas was hydrogen (0.5 bar). The injector and the flame ionization detector were at 225°C and 245°C, respectively.

#### 2.6.4. Neutral Lipid Molecular Analysis

Internal standards were added to the lipid extract (100 *μ*L from the total 400 *μ*L): 3 mg of stigmasterol, 1 mg of 1,3-dimyristine, 1 mg of cholesteryl heptadecanoate, and 5 mg of glyceryl triheptadecanoate. Neutral lipids were separated using a SPE column (Strata SI-1 Silica 55 *μ*m, 70 Å, 100 mg), washed with 2 mL of chloroform, crudely dissolved in 20 *μ*L of chloroform, applied on the cartridge, and eluted with 2 mL of chloroform. The organic phases were evaporated until dry and dissolved in 20 *μ*L of ethyl acetate. One *μ*L of the lipid extract was analysed by gas-liquid chromatography on a FOCUS Thermo Electron system using a Zebron-1 Phenomenex-fused silica capillary column (5 m × 0.32 mm i.d., 0.50 mm film thickness). Oven temperature was programmed from 200°C to 350°C at a rate of 5°C per min, and the carrier gas was hydrogen (0.5 bar). Profiles of neutral lipid molecular species were determined according to total acyl carbon number [32]. The injector and the flame ionization detector were operating at 315°C and 345°C, respectively.

### 2.7. Data Analysis

Data are expressed as mean ± SEM, and *n* represents the number of experiences. Statistical analyses were performed using a one-way analysis of variance (ANOVA) and Mann–Whitney *U* tests or ANOVA for repeated measures and subsequent Bonferroni's post hoc test. *P* < 0.05 was considered as statistically significant.

## 3. Results

### 3.1. Provinols Did Not Affect Contractile Response to Phe in both Aortas and SMA from ZF Rats

**Figure 1:**
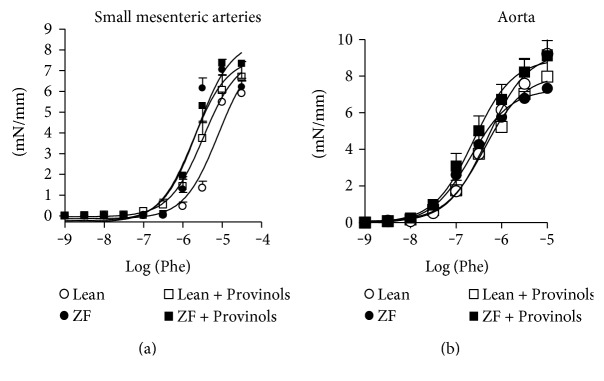
Provinols did not affect contractile response to Phe in aortas and SMA from obese ZF rats. Shown are concentration-response curves to phenylephrine (Phe) cumulative concentrations (M) of small mesenteric arteries (SMA, (a)) and rat aortic rings (b) from lean and ZF rats treated or not with Provinols. Values are expressed as mean ± SEM of millinewtons (mN) per mm of vessel length. *N* = 6 in each group.

The contractile response to a vasoconstrictor agonist (Phe) was investigated in both conductance (aorta) and resistance (SMA) arteries from ZF and lean rats with or without Provinols treatment. As shown in Figure 1(), there was no significant difference in the contractile response to Phe between all the groups in both the aorta and SMA. Due to the absence of differences in overall contraction between lean and ZF rats, the rest of the experiments were carried out on ZF rats only.

### 3.2. Effect of NO Inhibition on Vascular Reactivity in Aortas and SMA from ZF Rats

**Figure 2:**
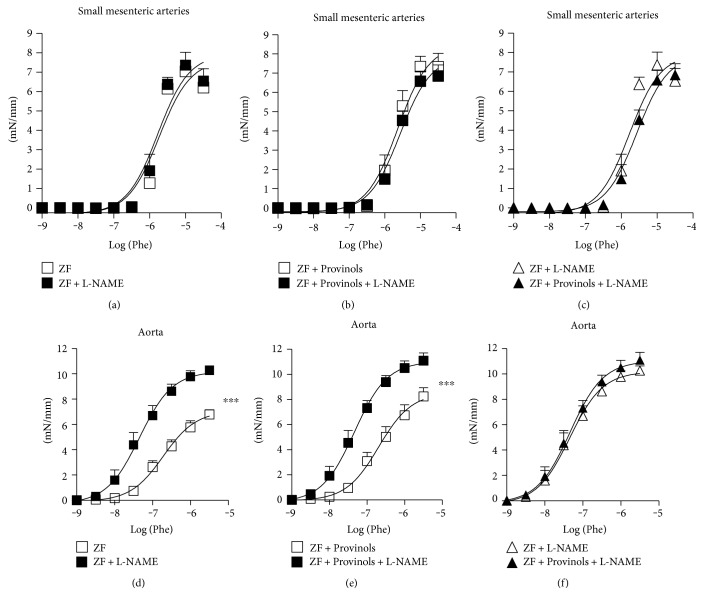
Effect of NO inhibition on vasoreactivity in aortas and SMA from obese ZF rats. Shown are concentration-response curves to phenylephrine (Phe) cumulative concentrations (M) in the presence of NO-synthase nonselective inhibitor (L-NAME) of small mesenteric arteries (SMA) (a–c) and rat aortic rings (d–f) from ZF rats treated or not with Provinols. Values are expressed as mean ± SEM of millinewtons (mN) per mm of vessel length. ^∗∗∗^*P* < 0.001 versus ZF rats.

To assess the contribution of NO levels in vasoconstriction of vessels from ZF and Provinols-treated ZF rats, NO synthases (NOS) were inhibited using L-NAME and then contraction in response to Phe was assessed in both the aorta and SMA. While the nonselective blockade of NOS did not modify contraction in response to Phe in SMA from both groups of rats (Figures 2(), 2(), and 2()), it potentiated to the same extent the contractile response to Phe in aortas from both ZF and Provinols-treated ZF rats (Figures 2(), 2(), and 2()).

### 3.3. Provinols Reduced the Involvement of COX-Derived Vasoconstrictor Metabolites in SMA from Obese Rats

**Figure 3:**
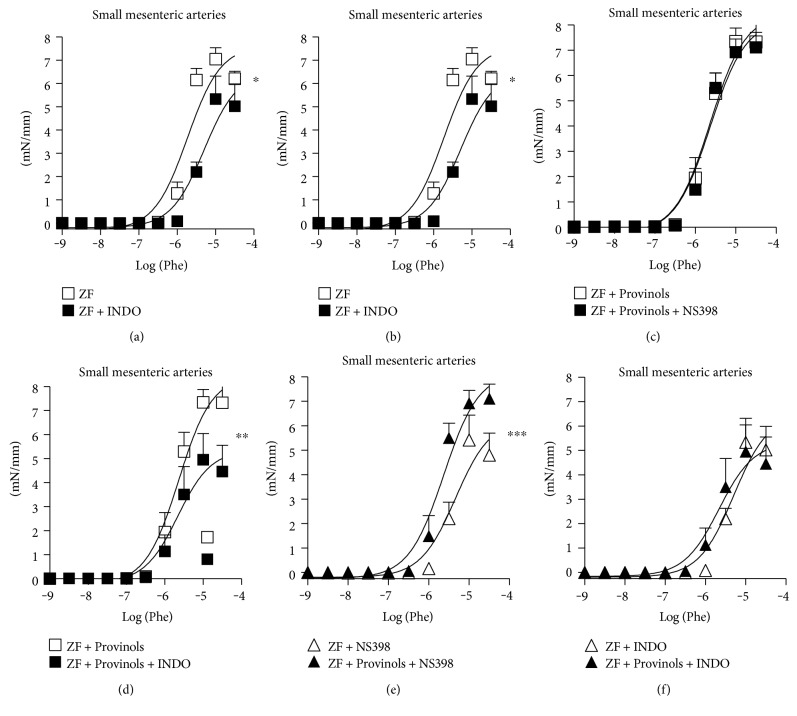
Provinols reduced the involvement of COX-derived vasoconstrictor metabolites in SMA from obese ZF rats. Concentration-response curves to phenylephrine (Phe) cumulative concentrations (M) in the presence of COX-2 inhibitor (NS398) or the nonselective COX inhibitor (indomethacin, INDO) of small mesenteric arteries (SMA) from ZF rats which received or not Provinols. *N* = 6 in each group. Values are expressed as mean ± SEM of millinewtons (mN) per mm of vessel length. ^∗^*P* < 0.05; ^∗∗^*P* < 0.01; ^∗∗∗^*P* < 0.001 versus ZF rats.

To figure out the relative contribution of vasoactive prostanoid derivatives in the contractile response to Phe in SMA, COX isoforms were blocked using selective and non-selective inhibitors. In ZF rats, both COX-2 (NS398) (Figure 3()) and nonselective COX (INDO) (Figure 3()) inhibitors reduced to the same extent the contractile response to Phe in SMA, suggesting the involvement of COX-2-derived vasoconstrictor metabolites in this contractile response. In Provinols-treated ZF rats, NS398 (Figure 3()) failed to affect the contractile response to Phe; however, indomethacin (Figure 3()) reduced contraction in SMA, suggesting the implication of non-COX-2-derived vasoconstrictor metabolites in this contractile response. Interestingly, when we compared the contractile response in the presence of inhibitors between the two groups, we observed that contraction was greater in vessels from Provinols-treated ZF rats in the presence of COX-2 inhibitor (Figures 3() and 3()), indicating a smaller contribution of COX-2-derived vasoconstrictor metabolites in the contractile response to Phe in SMA obtained from ZF rats which received Provinols compared to ZF rats.

### 3.4. Provinols Reduced the Involvement of COX-Derived Vasoconstrictor Metabolites in Aortas from Obese ZF Rats

**Figure 4:**
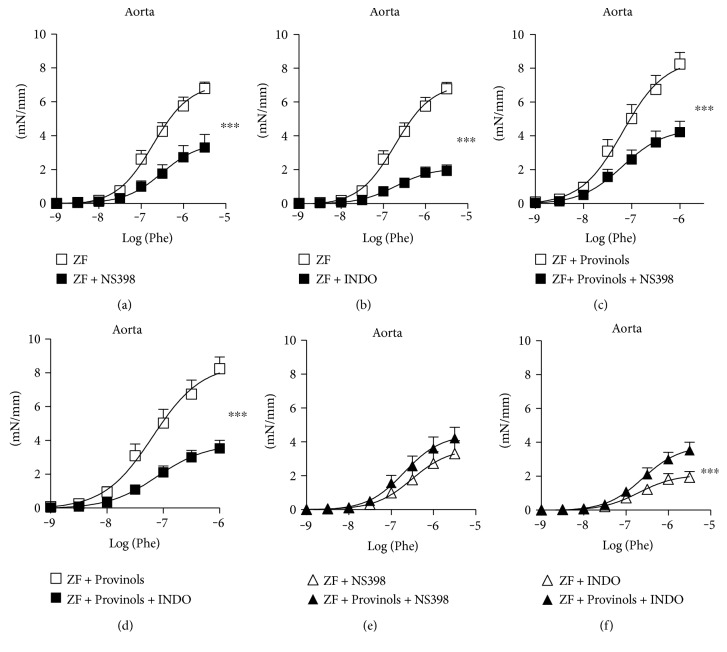
Provinols reduced the involvement of COX-derived vasoconstrictor metabolites in the aorta from obese ZF rats. Concentration-response curves to phenylephrine (Phe) cumulative concentrations (M) in the presence of COX-2 inhibitor (NS398) or the nonselective COX inhibitor (indomethacin, INDO) of aorta rings from ZF rats which received or not Provinols. Values are expressed as mean ± SEM of millinewtons (mN) per mm of vessel length. *N* = 6 in each group. ^∗∗∗^*P* < 0.001 versus ZF rats.

The relative contribution of vasoactive prostanoid derivatives in the contractile response to Phe was assessed in aortas by inhibiting COX isoforms. Both the nonselective inhibition of COX and the selective blockade of COX-2 reduced the contractile response to Phe in aortas from ZF rats; however, the reduction in contractile response was higher with INDO (Figures 4() and 4()), suggesting the participation of both COX-2- and non-COX-2-derived vasoconstrictor metabolites in the contractile response to Phe in ZF rats. Aortas from ZF rats which received Provinols exhibited a reduction in the contractile response to Phe in the presence of NS398 or INDO; however, the reduction was slightly higher in the presence of INDO, suggesting the involvement of mainly COX-2-derived metabolites in the aortas from the polyphenol group (Figures 4() and 4()).

The comparison of vascular contraction between aortas from both experimental groups in the presence of inhibitors showed no major differences in the presence of NS398, although vessels from the polyphenol group had a trend for a higher contractile response (Figure 4()); however, in the presence of INDO, the contraction in vessels from the polyphenol group was significantly greater (Figure 4()), suggesting a reduced involvement of non-COX-2-derived vasoconstrictor metabolites in aortas from Provinols-treated ZF rats.

### 3.5. Provinols Reduced COX-Derived Vasoconstrictor Metabolite Release in Aortas and SMA from Obese ZF Rats

**Figure 5:**
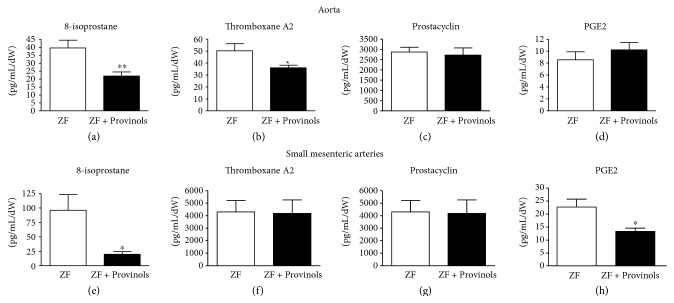
Provinols reduced COX-derived vasoconstrictor metabolite release in aortas and SMA from obese ZF rats. Concentration of COX derivatives thromboxane A2, prostaglandin E2 (PGE2), 8-isoprostane, and prostacyclin in the supernatants of the rat aorta and SMA from ZF rats treated or not with Provinols and stimulated with Phe (*n* = 6). The concentration of prostanoids is expressed as pg/mL/mg of dry weight (dw) tissue. ^∗^*P* < 0.05; ^∗∗^*P* < 0.01 versus ZF rats.

The functional studies suggest a reduced participation of COX-derived vasoconstrictor metabolites in both conductance and resistance arteries from Provinols-treated ZF rats. Indeed, in the aorta, the measurement of COX-derived metabolite production in vessels showed that the Provinols reduced the release of 8-isoprostane and TXA2, both vasoconstrictor metabolites, without affecting that of prostacyclin and prostaglandin E metabolites (Figures 5(a)–5(d)). In SMA, the Provinols reduced the production of vasoconstrictor metabolites 8-isoprostane and prostaglandin E, without modifying the release of prostacyclin and TXA2 metabolites (Figures 5(e)–5(h)).

Altogether, these data indicate that the Provinols reduced the participation of COX-derived vasoconstrictor agents in the contractile response to Phe in both aortas and SMA.

### 3.6. Provinols Modulated the Expression of COX Isoforms and NF-*κ*B in Vessels from Obese ZF Rats

**Figure 6:**
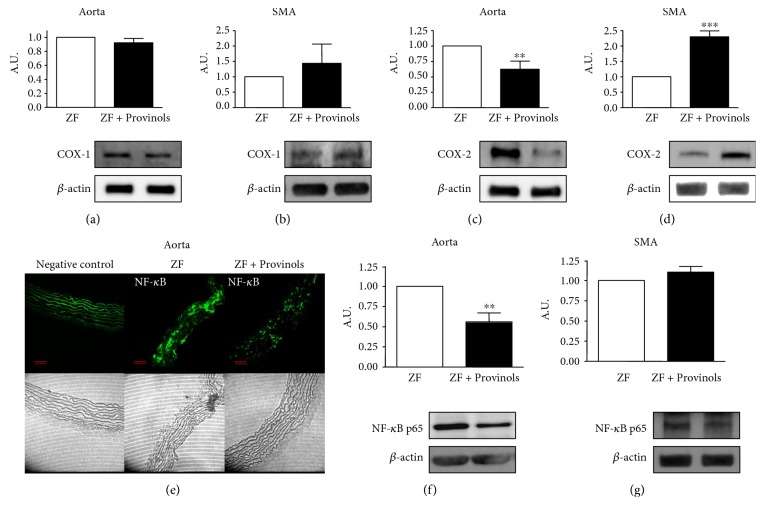
Provinols modulated the expression of COX isoforms and NF-*κ*B in vessels from obese ZF rats. Western blots showing the expression of cyclooxygenase- (COX-) 1 (a, b) and COX-2 (c, d) in the aorta and SMA from ZF rats treated or not with Provinols. NF-*κ*B expression in the rat aorta was assessed by confocal imaging (e) and western blot analysis (f). In (c) is shown NF-*κ*B expression in SMA by western blot analysis. Quantification of immunoblots' signal was done by densitometric analysis; *β*-actin loading control was included. Data are representative of four separate blots, and the densitometry values are expressed in arbitrary units (A.U.) as mean ± SEM. ^∗∗^*P* < 0.01; ^∗∗∗^*P* < 0.001 versus ZF rats.

Since Provinols could modulate the contribution of COX-derived agents in the contractile response of vessels from obese rats, the expression of COX-1 and COX-2 was therefore assessed. Results showed that Provinols did not affect the expression of COX-1, neither in the aorta nor in SMA (Figures 6(a) and 6(b)); however, it paradoxically affected the expression of COX-2 in aortas and SMA. While Provinols reduced COX-2 expression in the aorta, it enhanced its expression in SMA, (Figures 6(c) and 6(d)).

The transcription factor NF-*κ*B controls the transcription of proinflammatory enzymes such as COX-2; hence, its expression was assessed. The expression of NF-*κ*B was significantly reduced by Provinols within the aorta (Figures 6(e) and 6(f)); however, its expression did not change in SMA (Figure 6(g)).

### 3.7. Provinols Altered Phospholipid Profile in Carotids from Obese ZF Rats

An alteration in the activity of phospholipase A2, primarily responsible of prostanoid derivatives generation, may lead to an alteration of the lipid composition in vessels from ZF obese rats. Hence, the effects of Provinols on the distribution of membrane neutral lipids and fatty acids were analysed in carotid artery from ZF rats.

As shown in Table 1, the neutral lipid composition and fatty acid composition of total lipids from carotid tissues remained similar in Provinols-treated and control groups. Neither concentrations nor percent distribution of neutral lipid molecular species of triacylglycerides (TAG), diacylglycerides D (AG), nor cholesteryl esters were modified by Provinols (Table 1). Moreover, Provinols did not modify neither the concentration nor the distribution of fatty acid molecular species of saturated fatty acids (SAFA), monounsaturated fatty acids (MUFA), and polyunsaturated fatty acids (PUFA) (Table 2). However, even though phospholipid contents were similar in the two groups, Provinols led to an alteration of the phospholipid's profile. As shown in Table 3, Provinols significantly increased the contribution of phosphatidylethanolamine (PE) to total phospholipids (41.6 ± 6.4 nmol/mg and 50 ± 2.7 nmol/mg from nontreated and Provinols-treated ZF rats, respectively).

## 4. Discussion

Our findings reported in this study indicated that red wine polyphenols modulated the relative contribution of COX-derived metabolites in vasoreactivity in both conductance and resistance arteries from ZF rats, although without affecting the global contractile response of vessels. Indeed, Provinols could reduce the release of COX-derived vasoconstrictor agents from both types of arteries by affecting the expression and/or activity of COX enzymes as well as NF-*κ*B transcription factor. In addition, Provinols treatment altered the phospholipid molecular species distribution in carotid arteries from ZF rats. These data underscore the beneficial role of red wine polyphenols in modulating the contribution of inflammatory agents in the control of vascular tone in obese rats.

In a previous study, we showed that ZF rats had an impaired endothelium-dependent relaxation to acetylcholine compared to their lean controls [19]; however, the present study demonstrated that there was no difference in the contractile response to Phe between control and obese rats. In a study by Mingorance et al. [5], authors found that aortas and SMA from ZF rats had a lower response to Phe than lean animals, whereas ZF rats showed a diminished response to acetylcholine only in the aorta. The discrepancy between our findings and those from Mingorance et al. may be due to the age of animals used, 15 weeks in our study and 31 weeks of age in the other one. In addition, an exaggerated adrenergically mediated vascular reactivity has been also documented in aortic and SMA rings from obese rats [33, 34].

Although no difference was observed in the contractile response to Phe, we observed that Provinols treatment significantly modulated the relative contribution of COX-derived metabolites in vasoreactivity in both the aorta and SMA. Of note, SMA from ZF rats had more contribution from COX-2-derived vasoconstrictor metabolites in the contractile response to Phe compared to vessels from ZF rats fed a diet containing Provinols. Aortas from ZF rats had, however, more contribution from non-COX-2-derived vasoconstrictor agents in the contractile response to Phe in comparison to aortas from the polyphenol group. Consistent with these findings, the evaluation of prostanoid derivative release revealed that vasoconstrictor COX-derived agents were reduced in the Provinols group (8-isoprostane/TXA2 in the aorta and 8-isoprostane/prostanglandin E in SMA). Interestingly, previous studies showed an increased participation of COX-derived vasoconstrictor agents (i.e., TXA2) in vessels from ZF rats compared to lean controls [4, 5]. Both the aorta and SMA from the Provinols group had reduced levels of 8-isoprostane production, indicating a reduction of oxidative stress. Indeed, in contrast to classic prostaglandins, isoprostanes are formed by free radical-catalysed lipid peroxidation of arachidonic acid and cell membrane phospholipids and constitute thus a good marker for oxidative stress level. These observations are in line with our previous results which indicated that Provinols reduced superoxide anion release in aortas and SMA from ZF rats [19].

Findings from our study support previous reports demonstrating that polyphenols can regulate COX-2 expression and secretion [35, 36]. In this study, we observed that Provinols did not affect the expression of the constitutive COX-1 isoform in both the aorta and SMA; paradoxically, however, Provinols reduced COX-2 expression in the aorta and enhanced its protein expression in SMA. These results are not in apparent line with our functional findings or those of biochemical analysis of COX-derived metabolites release. The reduction in COX-2 expression in aortas may explain the reduction of the release of COX-derived metabolites. This is further corroborated by the reduction of NF-*κ*B transcription factor expression in aortas from the polyphenol group. The activation of NF-*κ*B is upstream of the synthesis of acute phase inflammatory mediators. Among the genes known to be positively regulated by NF-*κ*B is *COX-2* [37]. In the present work, we found that Provinols reduced NF-*κ*B in the aorta, but not in SMA, accounting potentially for reducing COX-2 expression. Previously, it has been shown that tea polyphenols inactivated phosphorylated forms of nuclear NF-*κ*B and reduced COX-2 expression in rat mammary tumours [38]. Furthermore, procyanidin extracts, a mixture of polyphenols, inhibited NF-*κ*B (p65) translocation in lipopolysaccharide-stimulated macrophages [39].

In SMA from rats treated with polyphenols, we observed an increase in COX-2 expression even though functional data and biochemical analysis indicated that these vessels have less contribution of COX-2-derived metabolites towards the contractile response to Phe. This paradoxical effect could be explained by a subtle effect of Provinols on the activity of COX enzymes and/or phospholipase A2 activity, which would contribute to the reduced release of COX-derived metabolites. The increase in COX-2 protein expression may therefore be a compensatory mechanism to the decrease in the enzyme's activity. Although the reduction in COX-derived metabolites caused by Provinols may suggest a reduction in COX activity, further studies are warranted to ascertain the effects of Provinols on COX enzymes' activity. Consistent with our findings, a previous study from Kane et al. [40] showed that red wine polyphenols reduced angiotensin II-induced COX-2 overexpression in rat vessels. Previous publications have also reported an inhibitory effect of red wine extract and tea polyphenols on COX-2 activity [20, 38]. However, a recent study evaluated the effect of several food polyphenols, including resveratrol, a component of red wine polyphenols, on the activity of COX-2 and reported that although these compounds could elicit the inhibition of COX–2, their potency was 100- to 1000-fold lower compared to known pharmacological inhibitors of COX-2 such as celecoxib and indomethacin [41].

Finally, we have shown that one focal effect of Provinols treatment was an alteration of phospholipid molecular species in carotid arteries from ZF rats. The increase in the PE contribution to total phospholipids suggested that Provinols treatment had a specific effect on phospholipid turnover because neutral lipids and fatty acid molecular species remain unchanged. Indeed, a reduction in PE degradation has been previously observed in platelets and liver microsomes due to inhibition of the secretory phospholipase A2 [42]. This result is suggestive of a reduction in the activity of phospholipase A2 in vessels from Provinols-treated rats especially when taken together with the reduction in the secretion of bioactive prostanoids.

In conclusion, we showed in this study that the global response to vasoconstrictor agonists was not altered in ZF compared to lean rats; however, red wine polyphenols could modulate COX expression and/or activity via a mechanism involving NF-*κ*B pathway. The present study provided evidence of a subtle regulation of vasocontractility both in conductance and resistance arteries by Provinols to maintain vascular tone within a physiological range. Such effect is linked to a paradoxical effect of Provinols on COX pathway. These data highlight further the beneficial role of red wine polyphenols in correcting cardiovascular disturbances associated with obesity and metabolic syndrome.

## Figures and Tables

**Table 1:** Comparison between neutral lipid molecular species from carotids in control and Provinols-treated rats.

Neutral lipids	ZF	ZF + Provinols
*Percent distribution of TAG*		
TAG C49	4.7 ± 0.3	3.8 ± 0.6
TAG C51	13.5 ± 0.8	12.1 ± 1.3
TAG C53	32 ± 0.9	31.4 ± 1
TAG C55	34.6 ± 1	35.77 ± 1.2
TAG C57	13.3 ± 0.7	14.6 ± 1.2
TAG C59	1.8 ± 0.4	2.4 ± 0.6
*Total TAG (nmol/mg)*	**4.68 ± 1.72**	**4.36 ± 1.35**
*Percent distribution of DAG*		
DAG 14–16	16.9 ± 11.6	12 ± 2.5
DAG 16-16	19.6 ± 6.7	14.4 ± 4.4
DAG 16–18	34 ± 9.2	43 ± 5.3
DAG 18-18	17 ± 14.7	19 ± 11.2
DAG18–20	12 ± 2.5	14 ± 0.3
DAG18–22	Nd	Nd
*Total DAG (nmol/mg)*	**0.10 ± 0.03**	**0.08 ± 0.03**
*Percent distribution of cholesteryl esters*		
C14 cholesteryl ester	Nd	Nd
C16 cholesteryl ester	15.1 ± 1.7	17.3 ± 3.8
C18 cholesteryl ester	17.9 ± 6	17.6 ± 5.1
C20:4 cholesteryl ester	67 ± 6.1	65.1 ± 8.3
C22 cholesteryl ester	Nd	Nd
*Total cholesteryl esters (nmol/mg)*	**1.8 ± 0.6**	**0.13 ± 0.03**
*Cholesterol (nmol/mg)*	**0.12 ± 0.04**	**1.6 ± 0.3**

Data are means ± SEM. Nd: not detected. Concentrations are expressed as nanomoles per mg of tissue wet weight (nmol/mg). Triacylglycerides (TAG) and diacylglycerides (DAG) families were defined according to their total number of carbon atoms (as described in Materials and Methods).

**Table 2:** Comparison between fatty acid (FA) molecular species from carotids in control and Provinols-treated rats.

FA	ZF	ZF + Provinols
*Total FA (nmol/mg)*	**14.59 ± 4.58**	**19.74 ± 6.43**
*Percent distribution of FA*		
SAFA	41.5 ± 2.4	40.9 ± 1.3
MUFA	38.1 ± 4.9	39.7 ± 1.4
PUFA	20.4 ± 2.6	19.4 ± 2.1
*Percent distribution of SAFA*		
14 : 0	3.3 ± 0.7	3.4 ± 1.3
16 : 0	72.6 ± 5.9	72.5 ± 2.7
18 : 0	22 ± 4.4	22.9 ± 3.6
20 : 0	1.1 ± 0.5	0.6 ± 0.2
24 : 0	1.1 ± 0.8	0.6 ± 0.2
*Percent distribution of MUFA*		
16 : 1 n-7	17.8 ± 0.8	17 ± 2.4
18 : 1 n-9	78.5 ± 4.3	82 ± 2
20 : 1 n-9	0.4 ± 0.1	0.4 ± 0.2
22 : 1 n-9	Nd	Nd
24 : 1 n-9	3.3 ± 4.5	0.6 ± 0.3
*Percent distribution of PUFA*		
18 : 2 n-6	53.1 ± 11.7	57.9 ± 9.3
18 : 3 n-6	Nd	Nd
18 : 3 n-6	2.1 ± 0.8	2.3 ± 0.6
20 : 2 n-6	1.3 ± 0.3	1.4 ± 0.4
20 : 3 n-6	Nd	Nd
20 : 3 n-6	1.7 ± 0.4	1.6 ± 0.4
20 : 4 n-6	38.5 ± 12	33.2 ± 9.2
20 : 5 n-3	Nd	Nd
22 : 5 n-3	Nd	Nd
22 : 2 n-6	Nd	Nd
22 : 6 n-3	3.3 ± 1	3.4 ± 1

Data are means ± SEM. Total fatty acids (FA) are expressed as nanomoles per mg of tissue wet weight. SAFA: saturated fatty acids; MUFA: monounsaturated fatty acids; PUFA: polyunsaturated fatty acids; Nd: not detected.

**Table 3:** Comparison between phospholipids (PL) from carotids in control and Provinols-treated rats.

Percent distribution of PL	ZF	ZF + Provinols
PE	41.6 ± 6.4	50 ± 2.7^∗^
PC	38 ± 5.7	33.9 ± 1.9
SM	13 ± 4.4	9.4 ± 1
PS	4.8 ± 1.2	4.3 ± 0.6
PI	2.6 ± 1.6	2.5 ± 0.4
PG	Nd	Nd
*Total PL (nmol/mg)*	**3.8 ± 1.3**	**3.6 ± 0.9**

Data are means ± SEM. Total phospholipids (PL) are expressed as nanomoles per mg of tissue wet weight. ^∗^Difference between groups at *P* < 0.05 (*n* = 5). PE: phosphatidylethanolamine; PC: phosphatidylcholine; SM: sphigomyeline; PS: phosphatidylserine; PI: phosphatidylinositol, PG: phosphatidylglycerol; Nd: not detected.
